# Dissemination and implementation research coordination and training to improve cardiovascular health in people living with HIV in sub-Saharan Africa: the research coordinating center of the HLB-SIMPLe Alliance

**DOI:** 10.1186/s43058-024-00599-4

**Published:** 2024-06-06

**Authors:** Emmanuel K. Tetteh, William Effah, Lisa de las Fuentes, Karen Steger-May, Charles W. Goss, David W. Dowdy, Mark D. Huffman, Makeda J. Williams, Veronica Tonwe, Geetha P. Bansal, Elvin H. Geng, Victor G. Dávila-Román, Treva Rice, Kenneth B Schechtman

**Affiliations:** 1 Institute for Informatics, Data Science and Biostatistics, Washington University School of Medicine, St. Louis, MO USA; 2 Center for Public Health Systems Science, Brown School, Washington University in St. Louis, St. Louis, MO 63130 USA; 3 Cardiovascular Division, Department of Medicine, Washington University School of Medicine, St. Louis, MO USA; 4 Department of Epidemiology, Johns Hopkins Bloomberg School of Public Health, Baltimore, MD USA; 5 The George Institute for Global Health, University of New South Wales, Sydney, Australia; 6 Center for Translation Research and Implementation Science, National Heart, Lung, and Blood Institute, National Institutes of Health, Bethesda, MD USA; 7 John E Fogarty International Center, National Institutes of Health, Bethesda, MD USA; 8 Division of Infectious Diseases, Department of Medicine, Washington University School of Medicine, St. Louis, MO USA

**Keywords:** Capacity building, Implementation science, Cardiovascular Health, Cardiovascular Disease, Hypertension, HIV/AIDS, HLB-SIMPLe

## Abstract

As global adoption of antiretroviral therapy extends the lifespan of People Living with HIV (PLHIV) through viral suppression, the risk of comorbid conditions such as hypertension has risen, creating a need for effective, scalable interventions to manage comorbidities in PLHIV. The Heart, Lung, and Blood Co-morbiditieS Implementation Models in People Living with HIV (HLB-SIMPLe) Alliance has been funded by the National Heart, Lung, and Blood Institute (NHLBI) and the Fogarty International Center (FIC) since September 2020. The Alliance was created to conduct late-stage implementation research to contextualize, implement, and evaluate evidence-based strategies to integrate the diagnosis, treatment, and control of cardiovascular diseases, particularly hypertension, in PLHIV in low- and middle-income countries (LMICs).

The Alliance consists of six individually-funded clinical trial cooperative agreement research projects based in Botswana, Mozambique, Nigeria, South Africa, Uganda, and Zambia; the Research Coordinating Center; and personnel from NIH, NHLBI, and FIC (the Federal Team). The Federal Team works together with the members of the seven cooperative agreements which comprise the alliance. The Federal Team includes program officials, project scientists, grant management officials and clinical trial specialists. This Alliance of research scientists, trainees, and administrators works collaboratively to provide and support venues for ongoing information sharing within and across the clinical trials, training and capacity building in research methods, publications, data harmonization, and community engagement. The goal is to leverage shared learning to achieve collective success, where the resulting science and training are greater with an Alliance structure rather than what would be expected from isolated and unconnected individual research projects.

In this manuscript, we describe how the Research Coordinating Center performs the role of providing organizational efficiencies, scientific technical assistance, research capacity building, operational coordination, and leadership to support research and training activities in this multi-project cooperative research Alliance. We outline challenges and opportunities during the initial phases of coordinating research and training in the HLB-SIMPLe Alliance, including those most relevant to dissemination and implementation researchers.

Contributions to the literature

- The Heart, Lung, and Blood Co-morbiditieS Implementation Models in People Living with HIV (HLB-SIMPLe) Alliance includes six late-stage implementation research studies to integrate hypertension and HIV care in people living with HIV in Botswana, Mozambique, Nigeria, South Africa, Uganda, and Zambia.
- The Alliance, including the Research Coordinating Center, supports research conducted at the unique intersection of HIV control practices, cardiovascular disease diagnoses and management, health economics, and dissemination and implementation science to enhance shared learning in the based practices for translating evidence-based practices into routine practice across varying health care ministries and healthcare settings where the comorbid burden of HIV and cardiovascular diseases is significant.

## Background

The global public health response to Human Immunodeficiency Virus (HIV) disease has transformed the epidemic over the past forty years. In low- and middle-income countries (LMICs), antiretroviral therapy is now widely accessible and has contributed to controlling disease progression in most patients and reducing disease transmission [1]. Furthermore, health systems, human resources, supply chains, and information infrastructure created to provide long-term care for HIV-affected people provides an opportunity to leverage the delivery of widely affordable and efficacious therapies for chronic, non-communicable diseases (NCD) among people living with HIV (PLHIV). The Heart, Lung, and Blood Co-morbiditieS Implementation Models in People Living with HIV (HLB-SIMPLe) Alliance is funded by the National Heart, Lung, and Blood Institute (NHLBI) and the Fogarty International Center (FIC) of the United States National Institutes of Health (NIH), through Funding Opportunity Announcements RFA-HL-20-025 and RFA-HL-20-026 [2, 3]. The central goal of the Alliance is to conduct late-stage dissemination and implementation research to deliver evidence-based strategies for the diagnosis, treatment, and control of comorbid cardiovascular diseases in PLHIV. HLB-SIMPLe seeks to catalyze change through research on implementation strategies that optimally and sustainably incorporate heart, lung, and blood disorder management into health systems developed for HIV care among high-burden populations. The HLB-SIMPLe Alliance research projects are funded through a biphasic (UG3/UH3) funding mechanism through which planning, feasibility, and pilot studies are conducted during the initial UG3 phase and a larger clinical trial is conducted during the UH3 phase.

The Alliance is comprised of leaders in dissemination and implementation (D&I) conducting research in six participating countries: Botswana, Mozambique, Nigeria, South Africa, Uganda, and Zambia. Each of the participating research teams is independently funded and responsible for the conduct of its clinical trial. The HLB-SIMPLe Alliance Research Coordinating Center (RCC) based at Washington University in St. Louis is responsible for providing resources that promote shared learning across and among the Alliance. The RCC’s resources support efforts related to data harmonization, human subjects and regulatory assistance, data management and analysis, site monitoring, communications, and operational and scientific capacity building, especially in the realm of dissemination and implementation research. The RCC also collaborates with the Federal Team to manage a global implementation research data safety and monitoring board (DSMB). The purpose of this paper is twofold: 1) to describe the HLB-SIMPLe Alliance and the coordination of research and training activities of the RCC, and 2) to highlight challenges and opportunities of the HLB-SIMPLe Alliance during the initial phases of coordinating research and training in the HLB-SIMPLe Alliance.

### Overview and structure of HLB-SIMPLe Alliance

**Figure 1:**
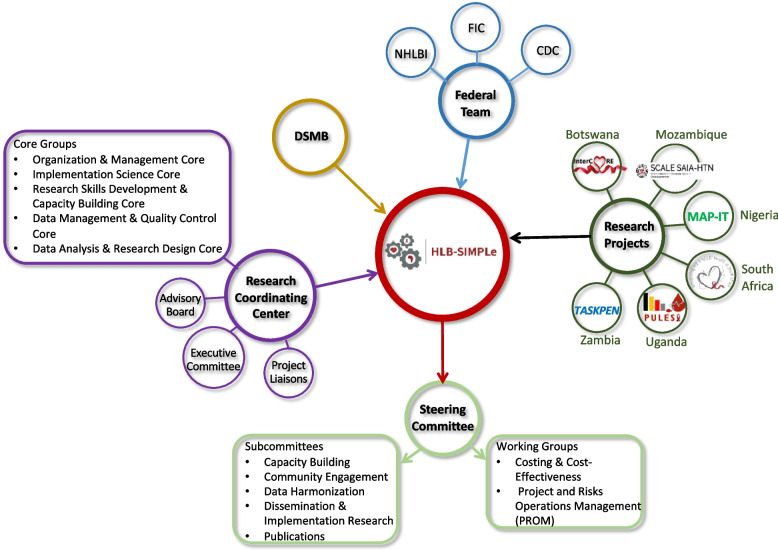
**Fig. 1**

The Alliance consists of six different, although thematically related, research projects that are individually funded by NHLBI and the FIC using a biphasic, milestone-driven UG3/UH3 mechanism. This funding mechanism requires research teams to develop and pilot their interventions in the first two years of the funding period (formative or UG3 phase), followed by three years of the intervention roll-out and implementation (implementation or UH3 phase). It is a cooperative agreement award that includes substantial NIH scientific and programmatic involvement in project activities. The RCC provides coordination and support to research projects during the entire five-year period of the Alliance [2, 3]. The governance network of the Alliance is illustrated in Fig. 1 with further descriptions of each group below.

### Research projects

The six research projects and their corresponding countries are listed in Table 1. All six HLB-SIMPLe research projects conduct implementation science research using cluster randomized trials, and all trials have chosen to focus on hypertension in PLHIV.

**Table 1:** HLB-SIMPLe Alliance Countries, Research Projects, and Trial Registrations

Trial Registration Number	Country – Project Name	Intervention	Study Design	Study Outcome Measures
NCT05414526	Botswana – Integrating Hypertension and Cardiovascular Disease Care into Existing HIV Service Package in Botswana (InterCARE)	The InterCARE package includes: a tailored inter-professional training curriculum on management of Cardiovascular Disease (CVD) risk and HIV; adaptation of the existing electronic health records; and adoption of a treatment partner support system.	Cluster randomized trial in 14 clinics. 7 clinics will receive the InterCARE package while the remaining 7 clinics will receive the local standard of care	Primary: RE-AIM assessment of InterCARE package (reach, effectiveness, adoption, implementation fidelity and maintenance). Secondary: Implementation feasibility, acceptability, CVD risk factor % change.
NCT05002322	Mozambique – Scaling Out and Scaling Up the Systems Analysis and Improvement Approach to Optimize the Hypertension Diagnosis and Care Cascade for HIV-infected Individuals (SCALE-SAIA HTN)	The SAIA-HTN implementation strategy uses an iterative, five-step process applied at the facility level to give clinic staff and managers a systems view of cascade performance, identify priority areas for improvement, discern modifiable solutions, and test workflow modifications.	Stepped wedge design with random assignment of two districts to each of the three nine-month intervention waves, to reach 6 districts in Maputo Province, Mozambique	Effectiveness outcomes: *Individual clinical* (controlled HTN and viral load suppression) *Service-level* (HTN screening, diagnosis, treatment and medication adherence). Other outcomes: RE-AIM assessment of SAIA-HTN strategy (reach, adoption, implementation, maintenance), and cost-effectiveness
NCT05031819	Nigeria – Managing Hypertension Among People Living with HIV: an Integrated Model (MAP-IT)	A nurse-led task-strengthening program, which includes CVD risk assessment; medication initiation and titration; lifestyle counseling and patient referral to physician care for complex cases of HTN in PLHIV.	A stepped wedge cluster randomized control trial across 30 primary healthcare centers	Primary: Adoption of task-strengthening program. Secondary: Blood pressure control, rate of adoption and implementation fidelity
NCT05846503	South Africa – Integrating HIV and Heart Health in South Africa (iHEART-SA)	iHEART-SA intervention comprises: Information management systems; task shifting; audit and feedback; healthcare worker education and training; patient education and support	Type 2 hybrid cluster randomized stepped wedge effectiveness-implementation across 9 primary healthcare centers	Primary: Difference in mean systolic BP between intervention and control groups. Secondary: Implementation fidelity, adoption, maintenance, satisfaction, cost-effectiveness of iHEART-SA intervention.
NCT05609513	Uganda – Strengthening the Blood Pressure Care and Treatment Cascade for Ugandans Living with HIV – Implementation Strategies to Save Lives (PULESA)	HTN-BASIC package: provision of diagnostic equipment and evidence-based antihypertensive drugs HTN-PLUS package: hypertension training; differentiated service delivery; remote patient monitoring; Performance Improvement Program, in addition to HTN-BASIC package	Stepped Wedge Cluster Randomized Control Trial. 16 clinics will be randomized to receive the HTN-BASIC package only or the enhanced HTN-PLUS intervention.	Primary: Blood Pressure (BP) control (% with BP < 140/90) Secondary: Scalability, sustainability, cost-effectiveness of PULESA packages
NCT05005130; NCT05950919	Zambia – Task-shifted Adaptation of the WHO-PEN Intervention to Address Cardio-metabolic Complications in People Living with HIV in Zambia (TASKPEN)	The TASKPEN package includes; adapted WHO-PEN protocols, algorithm, & training materials; access to cardio-metabolic condition screening & laboratory monitoring; Noncommunicable disease (NCD)-focused electronic medical record module; Integrated NCD/HIV care (“one stop shop” for services); strengthened NCD medication supply chain, including multi-month dispensing.	Type II Hybrid Effectiveness-implementation Stepped Wedge Trial across 12 sites	Primary: Proportion of participants with both HIV viral suppression and control of hypertension, diabetes and tobacco use. Secondary: Implementation fidelity, adoption, reach, cost-effectiveness, acceptability of TASKPEN package.

#### InterCARE (Botswana)

The InterCARE study aims to integrate evidence-based interventions for hypertension and Cardiovascular Disease (CVD) risk management into HIV care. It uses a type 2 hybrid design to measure the effectiveness of a multi-component intervention package (training and coaching of healthcare providers; modification of electronic health records; provision of basic equipment; and education and counseling for patients and using treatment partners) in improving treatment uptake and success rates in the hypertension cascade among HIV and hypertension [4].

#### SCALE-SAIA HTN (Mozambique)

This project plans to scale out a multi-component implementation strategy called Systems Analysis and Improvement Approach (SAIA) to standardize and improve hypertension care within existing HIV services in Mozambique. The study focuses on using district health supervisors to deliver the intervention and expanding it to additional provinces [5, 6].

#### MAP-IT (Nigeria)

Collaborating with the federal ministry of health and community organization, this step-wedge randomized control trial integrates a nurse-led Task-Shifting Strategy for HTN control (TASSH) program for hypertension control within HIV care, using a practice facilitation strategy. The TASSH program includes CVD risk assessment, medication initiation and titration, lifestyle counseling and patient referral to physician care for complex cases of HTN [7, 8].

#### iHEART-SA (South Africa)

This study utilizes a type 2 hybrid cluster randomized stepped-wedge effectiveness-implementation design to introduce the iHEART-SA intervention model across 9 primary healthcare centers [9]. The iHEART-SA intervention model is designed to enhance hypertension screening and management. It incorporates various strategies, such as an information management system, task shifting, audit and feedback, healthcare worker education and training, as well as patient education and support.

#### PULESA (Uganda)

This study utilizes a stepped-wedge cluster randomized trial to assess the effectiveness of implementation strategies in strengthening HTN care in PLHIV in urban and peri-urban clinics in Uganda [10]. The clinics are randomized to receive either the HTN-BASIC package or the enhanced HTN-PLUS intervention package. The HTN-BASIC package provides diagnostic equipment and evidence-based antihypertensive drugs at no cost. The HTN-PLUS package, in addition to the HTN-BASIC, offers hypertension training, differentiated service delivery, remote patient monitoring, and a Performance Improvement Program.

#### TASKPEN (Zambia)

This project focuses on integrating the World Health Organization Package of Essential Noncommunicable Disease (NCD) Interventions [11] into routine HIV care, emphasizing task shifting to nurses and community health workers, and providing integrated NCD/HIV care (a “one-stop shop” for services). It aims to reduce cardiovascular disease risk and improve HIV and NCD care outcomes [12].

### Federal team

The United States Government Federal Team includes program officials, project scientists, grants management officials, and clinical trials specialists from the NHLBI and FIC, with engagement from the Centers for Disease Control and Prevention (CDC). The Alliance uses a cooperative agreement as its administrative and funding instrument, characterized as an ‘assistance’ mechanism rather than an ‘acquisition’ mechanism [2, 3]. This arrangement denotes that the Federal Team has significant programmatic involvement with the awardees throughout the project’s execution. Within this cooperative agreement, the NIH’s purpose is to actively support and collaborate with the members of the seven cooperative agreements, cultivating a partnership, rather than taking on a directive role.

Federal Team members are represented on the Steering Committee, all subcommittees, and contribute to the planning of Alliance-wide meetings. The team provides perspective and expertise on all emerging issues and discussions. A project scientist from the Federal Team is assigned to each of the six research projects to function as an imbedded member of the research team providing scientific support during the planning and execution of the clinical trial and to facilitate collaboration with other NIH-supported research resources. Additionally, the program and grants management officials, in coordination with the DSMB, were responsible for the administrative review and approval of transitions from the formative (UG3) to implementation (UH3) phases of the research projects.

### Data and Safety Monitoring Board (DSMB)

A DSMB convened by the NHLBI in consultation with the RCC includes members who are experts in multicenter trials, global health, health economics, dissemination and implementation research, biostatistics, and research on HIV and heart, lung, and blood disorders. The DSMB reviews research projects annually to monitor the safety of Alliance research project participants, ethical conduct of research, assess study performance and data validity and integrity.

### Steering committee, subcommittees, and working groups

The roles and responsibilities of the Steering Committee primarily include formulating, implementing, and reviewing all Alliance-wide policies and procedures and establishing its scientific agenda. This committee seeks to identify opportunities for new collaborations and allocate shared resources for projects across awardees while coordinating data sharing and collaborative research efforts among awardees.

#### Leadership structure

The leadership structure of the Steering Committee, as with most of the subcommittees in the Alliance, consists of a chair and vice chair. The chair and vice chair of the Steering Committee and the subcommittees must be a representative of one of the research projects. RCC investigators provide administrative support to the chair and vice chairs. The RCC developed a template that provides a standardized structure for all subcommittee charters. Using that template, the RCC has assisted Steering Committee and subcommittees in developing charters that define features that include structures, membership, responsibilities, and voting procedures. The roles and activities of subcommittees and working groups are explained below.

#### Capacity building subcommittee

The Capacity Building Subcommittee is tasked with creating a curriculum and maintaining an inventory of training programs, educational materials, and other resources. It develops and implements a plan for sharing training materials across research projects, while developing metrics to evaluate trainee and program success. This subcommittee is the channel through which grant writing workshops and training programs, mock study sections, and small research projects are awarded to Alliance trainees and other early-career investigators. The activities supported by this subcommittee, especially, are in keeping with FIC’s strategic plan goals of building the research workforce in LMICs.

#### Community engagement subcommittee

The Community Engagement Subcommittee is the vehicle by which the Alliance operationalizes community engaged research in the context of dissemination and implementation science and facilitates the sharing of best practices and lessons learned for community engaged research within the HLB-SIMPLe Alliance and beyond. The committee is guided by the nine principles of Community-Based Participatory Action Research [13, 14].

#### Data harmonization subcommittee

An Alliance with multiple distinct research projects such as HLB-SIMPLe presents unique opportunities to create, harmonize, and analyze data that are common to multiple projects. These opportunities range from determining what data elements can and should be harmonized for joint statistical analysis, to developing strategies to maximize commonality across research projects. The primary objectives of the Data Harmonization Subcommittee are to develop strategies to maximize commonality of clinical data, implementation outcomes, and cost effectiveness measures. Additionally, this subcommittee works with the RCC to facilitate the statistical analysis of common data and produce generalizable learning across Alliance projects. Operationally, the Data Harmonization Subcommittee works with the Data Management and Quality Control Core of the RCC to transfer data elements from the six clinical trials to the RCC with the objective of merging into a single database using international data quality standards, such as the Observational Medical Outcomes Partnership Common Data Model [15, 16]. The harmonized data will be analyzed either by project biostatisticians and data analysts or by the RCC.

#### Dissemination and implementation science subcommittee

The Dissemination and Implementation Science Subcommittee formulates shared conceptual areas regarding implementation strategies and their mechanisms to inform potential for shared measurement of both qualitative and quantitative data. The subcommittee develops strategies to maximize commonality of the selected metrics (i.e., contextual factors, implementation outcomes) across Alliance projects where appropriate and as prioritized by the subcommittee. Additionally, it provides opportunities for peer-to-peer learning and sharing of dissemination and implementation research measurement methods and tools, and leverages members’ expertise, including that of the RCC, to provide methodological insights via consultations.

#### Publications subcommittee

The Publications Subcommittee is tasked with developing, coordinating, and executing policies, procedures, and guidelines for cross-project Alliance publications and presentations. It employs policy mechanisms that promote collaborative publications and presentations that highlight the focus of HLB-SIMPLe on implementation science in LMICs. The responsibilities of the Publications Subcommittee include: 1) reviewing multi-project manuscripts and presentations to ensure the accuracy of the publications’ content, consistency across publications, and identifying RCC resources necessary for project completion; 2) developing approaches that prioritize publications so resources are used efficiently; 3) establishing and monitoring dissemination strategies for publications (i.e., reports, scientific papers, journal articles, special journal issues, press releases, posters, other presentations); 4) establishing authorship policies and mechanisms for preventing and resolving authorship disputes; 5) creating a database of deadlines for relevant society meetings to facilitate abstract preparations; and 6) promoting and mentoring early-career investigators and trainees as authors. Additionally, the Publications Subcommittee assists Alliance projects in cataloging procedures, tools, products, activities, care pathways, and policies that enhance public health and well-being, employing the Translational Science Benefits Model framework [17].

#### Costing and health economics working group

LMICs are facing an escalating burden of heart, lung, and blood disorders among people with HIV. In implementing interventions to ameliorate this burden, evaluation of cost, cost-effectiveness, and budget impact are increasingly important. The Costing and Health Economics Working Group of the HLB-SIMPLe Alliance takes a coordinated approach to evaluating the cost and cost-effectiveness of these interventions. The responsibilities of the working group include: 1) developing a “minimum” set of standardized instruments for collecting data on costs in participating sites; 2) ensuring that best practices regarding costing methods are shared across sites; 3) providing technical support to sites that do not have internal health economics capacity; 4) organizing an annual health economics “summit” that includes didactic material, workshops, and communications across sites; 5) advancing the science of economic evaluation of integrated HIV-hypertension care through working papers and scoping reviews; and 6) building global capacity for health economics through North-South and South-South “pods” that meet on a monthly basis. Through these activities, the Costing and Health Economics Working Group seeks to leverage the ongoing health economics efforts at each of the six sites to create generalizable and actionable new knowledge, to create global research capacity, and to advance the field of economic evaluation of integrated health systems in LMICs.

#### Project and Risks Operations Management (PROM) working group

The Project and Risks Operations Management (PROM) Working Group employs strategies to identify, assess, and mitigate risks that could impact the success of the studies or projects. The core aims of the PROM Working Group are to facilitate the preparation of DSMB reports, uncover opportunities to share risk management plans for fostering continuity of research in the face of epidemics, civil unrest, and natural disasters; to share successes and challenges across projects; and to facilitate RCC support for all Alliance projects as needed.

### The HLB-SIMPLe Research Coordinating Center (RCC)

The RCC provides scientific and organizational coordination to ensure that the Alliance successfully creates rigorous knowledge related to dissemination and implementation science while also advancing shared learning to improve cardiovascular health in PLHIV. Through multifaceted research support and Alliance-wide capacity building activities, the RCC coordinates the Alliance to enhance opportunities for information sharing, training, and capacity building. The goal is to leverage shared learning as a means to achieve collective success, where the resulting science and training are greater with an Alliance structure rather than what would be expected from isolated and unconnected individual research projects. The structure of the RCC includes an external Advisory Board, Executive Committee, project liaisons and core groups which work closely with committees, subcommittees and working groups within the Alliance to provide administrative and scientific support towards achieving the intended goals and performance objectives of the Alliance.

#### Advisory board

An Advisory Board of four internationally recognized experts in implementation science, global health, NCD prevention, and HIV management was empaneled to provide recommendations, advice, support, and objective review of RCC activities. Members include: Ross Brownson, Ph.D. – Professor of Public Health at Washington University in St. Louis; Geoffrey Curran, Ph.D. – Professor at University of Arkansas for Medical Sciences; Eugene Mutimura, Ph.D. – Executive Secretary of the National Council for Science and Technology of Rwanda; and William Powderly, M.D. – Professor of Medicine at Washington University School of Medicine. The Board meets annually and is available for additional consultation as needed. The responsibilities of the Advisory Board are to advise the RCC regarding its research coordination and training, and to provide guidance on how the RCC can support the Alliance mission of providing a collaborative infrastructure that will facilitate the conduct of late-stage implementation research strategies to optimally and sustainably deliver proven-effective prevention and treatment interventions for hypertension in PLHIV.

#### RCC executive committee

The RCC principal investigators and two co-investigators meet weekly to discuss the administration of and to set operational and scientific priorities for the RCC to ensure that it meets its strategic targets and milestones. The executive committee is additionally responsible for decision making related to budgets, staffing, and communication to research project PIs and other members of the Federal Team.

#### Project liaisons

Each Alliance project is assigned one RCC research team member to serve as Project Liaison, functioning as a primary contact for each funded project to connect to and encourage engagement of project team members with Alliance resources, including scientific support when needed. Project Liaisons meet regularly (generally weekly to monthly) with project research teams, program scientists, clinical trials specialist, and program officials to review clinical trial progress. Additionally, Project Liaisons meet bi-monthly with RCC leadership to share updates on project progress with the objective of identifying programmatic opportunities for scientific and administrative support and capacity building to benefit individual projects and the Alliance as a whole. Liaisons also prepare periodic reports that track study progress and itemize successes and challenges.

### Core functions and activities of the RCC

The RCC has four core functions: 1) to oversee management, organizational, and communication activities, 2) to collaborate with and provide support for all research projects as they develop and implement the scientific and operational details of their protocols, 3) to support dissemination and implementation science research activities, and 4) to support and ensure the implementation of all research capacity building activities. These core functions and activities of the RCC are summarized in Table 2 below.

**Table 2:** Core Functions and Activities of the HLB-SIMPLe RCC

Core Functions	Activities
1. Oversee management, organizational, and communications activities	• Communications and Resource Sharing • Face-to-Face Meeting Logistics • RCC Milestones Reporting • Feedback Surveys and Evaluations
2. Provide Support for Alliance Projects	• Expert Consultant Support (External) • Data Management Support • Regulatory Research Site Monitoring
3. Support D&I Science Research Activities	• Implementation Research Frameworks • Implementation Strategies Reporting and Specification • Transportability Analysis
4. Capacity Building and Strengthening	• Develop Educational Materials to be Used Broadly by the Alliance • Co-organize and Co-lead Annual In-Person Symposia • Small Research Projects (SRPs) • Capacity Building Site Visits

### Core function 1: perform management, organizational, and communications activities

The activities of this core function of the RCC are implemented structurally through an Organization and Management Core that is responsible for the four major activities outlined below.

#### Communications and resource sharing

The RCC facilitates communication among Alliance participants, community collaborators, and other implementation science and global health researchers by providing, organizing, and managing multi-media communications platforms that include a website (www.hlbsimple.org) for resource-sharing, and secure communication and collaboration platforms to conduct business such as phone and video conferences, discussion groups, committee and subcommittee meetings, and meeting documentation and recordings. This Core also regularly publishes electronic newsletters for Alliance members that feature announcements, discussion of best practices, links to enduring materials from annual meetings and workshops, notices of funding opportunities, spotlights of Alliance members, and other newsworthy information. This core is also tasked with providing training on research communication to a cohort of communication champions at research projects.

#### Face-to-face meeting logistics

The Organization and Management Core at the RCC collaboratively shares the responsibility with local project teams for coordinating and planning all logistical aspects of Alliance-wide in-person meetings. This includes tasks ranging from selecting venues to conducting cost negotiations and managing contracts, facilitating agenda planning including speaker engagement, trainee presentations, poster sessions, and workshops. The Core is also responsible for planning and executing in-person capacity building site visits to research project countries.

#### RCC milestones reporting

This Core works within the RCC and in collaboration with the Federal Team to establish milestones, identify the responsible individuals, set timelines for delivery, and ensure milestone reports are received and appropriately stored. This Core also issues annual reports documenting RCC activities for the Advisory Board, NHLBI, and Alliance members.

#### Feedback surveys and evaluations

The RCC conducts feedback surveys and evaluations of training programs, workshops, and annual Alliance meetings. These surveys and evaluations serve to evaluate the performance of trainings and workshops using the Kirkpatrick’s evaluation model [18] and to gather valuable insights used to improve satisfaction with Alliance activities.

### Core function 2: provide technical support for Alliance projects

#### Expert consultant support

The RCC has identified consultants from outside the RCC who have in-depth supplemental expertise in specific technical areas such as health economics (e.g., costing and cost effectiveness). As an example, the RCC received supplementary funding to collect cost data in the two HLB-SIMPLe research projects that did not initially include health economics in their research plan, to advance the science of costing implementation of health interventions in LMICs, and to strengthen health economics capacity throughout the Alliance and more broadly.

#### Data management support

One member of the Data Management and Quality Control Core is assigned to work with each of the six research projects. The assigned data management expert supports the teams as data management and quality control systems are developed and implemented with an additional objective of identifying opportunities for cross-project analysis of harmonized data.

#### Regulatory site monitoring

The RCC conducts annual virtual regulatory site monitoring to support and ensure compliance with local and international regulations. Guidance and opportunities for training and certification in the protections of human subjects are provided by the RCC upon request from the research teams.

### Core function 3: support D&I science research activities

Central to the RCC and to the mission of this Alliance are crosscutting Dissemination and Implementation Science Core activities. These activities include the use of implementation research frameworks, a careful definition of implementation strategies and contexts, and transportability analyses. These three core pursuits are delineated below.

#### Implementation research frameworks

Leveraging the RCC’s experience in implementation theories, frameworks, and models, the Disseminations and Implementation Science core offer the opportunity to work with research sites to ensure that the selected implementation frameworks enhance conceptualization and well-defined constructs that yield theoretically sound research, if needed. For instance, RE-AIM has been considered as a shared framework for planning and evaluating intervention implementation across the research projects [19].

#### Implementation strategies

Translating evidence-based interventions into routine systems of care requires multi-level implementation strategies that are feasible, appropriate, acceptable, effective, affordable, and sustainable [20]. Proctor and colleagues recommend reporting the following elements: actors (who does what), action (the procedure, e.g., a workshop), dose (length of procedure), temporality (frequency of procedure), targets (intended audience), implementation outcomes (e.g., feasibility, acceptability), and the theoretical justification for the selected strategy [21]. In a systematic review of dissemination and implementation research in PLHIV, Hickey and colleagues found that only about one-half of published research studies reported all recommended elements [22], highlighting a need for improved reporting and harmonization. In view of this, the Dissemination and Implementation Science Core of the RCC works collaboratively with the Dissemination and Implementation Science Subcommittee to advise and provide guidance to research teams in tracking and reporting implementation strategies, using the 6 practical recommendations suggested by Lengnick-Hall and colleagues [23].

#### Transportability analysis

Transportability analysis is a mathematically grounded theory that formalizes conditions under which results of a study conducted in one setting can be applied to another [24]. Briefly, transportability theory shows that to apply results of a study to a new population, one must account for characteristics that: 1) differ between the two populations, and 2) modify the effect of the intervention under study. The Implementation Science Core of the RCC will work with the Dissemination and Implementation Science subcommittee of the Alliance to pursue novel approaches for assessing both external validity and estimation.

### Core function 4: capacity building and strengthening

The RCC is responsible for capacity building in multiple domains that include human subjects research, biostatistics, and training targeting early-career investigators (e.g., manuscript and grant writing). A recent NHLBI workshop identified two fundamental capacity-building opportunities – first, to build guidelines to engage trainees in dissemination and implementation research; and second, to create opportunities for established noncommunicable disease researchers in LMICs to learn about dissemination and implementation research [25]. With an understanding of these opportunities, the objective of the Research Skills Development and Capacity Building Core is to build infrastructure and training programs that support the development of multidisciplinary research skills related to HIV and comorbid heart, lung, and blood disorders in the context of D&I research. The specific activities of this core are outlined below.

#### Develop educational materials to be used broadly by the Alliance

The RCC has developed educational materials in biostatistics, grant writing, communications, costing and cost-effectiveness, and dissemination and implementation science to build research capacity in Alliance project institutions and countries. This educational platform provides opportunities for educational resources, interdisciplinary research, and a mentoring network. The RCC is also in the process of creating course materials that cover topics related to heart, lung, and blood disorders, including community engagement and implementation research for managing non-communicable diseases (NCDs), which will be incorporated into a Massive Open Online Course (MOOC) available online at www.hlbsimple.org/mooc. The objective of this initiative is to engage a broader audience, including healthcare researchers, administrators, policymakers, and other collaborative partners, providing them with an introduction to how implementation research can be applied within the context of NCD care.

#### Conduct annual in-person symposia

In collaboration with the NIH and Alliance research teams, the RCC co-organizes and co-leads 2-3-day annual in-person Alliance-wide symposia with the active participation of scientific experts, trainees, and Federal Team. Symposia topics include but are not limited to dissemination and implementation science, HIV, heart, lung, and blood disorders, biostatistics, and health economics; data and technology sharing; identifying collaborative training and research opportunities; optimizing research/training resources; workshops and round-table discussions; and trainee presentations of ongoing research.

#### Small Research Projects (SRPs)

The RCC provides funding for small research projects (SRPs) issued to trainees and other early-career investigators at the six Alliance research projects. The aims of the SRPs are: 1) to teach critical grant writing skills as an initial step to research independence; 2) to provide financial support for trainee/early-career investigator-proposed research projects; and 3) to facilitate the generation of pilot data in support of a subsequent research grant application (e.g., Fogarty International Center’s K43 Emerging Global Leader Award). All SRPs must focus on dissemination and implementation research topics or healthcare needs relevant to HLBS research priorities and populations living in LMICs.

#### Capacity building site visits

Members of the RCC travel to research project countries for research capacity building site visits. These visits help clarify the needs of the sites to effectively execute their research projects; offer the RCC crucial insights regarding local context that affect the trial design, conduct, and data generation; and provide opportunities to network with local collaborators, including patient groups, university leaders, implementing partners, and government officials. During these visits, early-career investigators present their research findings, fostering generative conversations and the exchange of insights. Other training programs and relevant resources are also discussed to support their research career development.

### Research coordinating center challenges and opportunities

#### Understanding implementation context

One of the significant scientific questions that besets the Alliance, as well as the field of implementation science as a whole, is the challenge of understanding context of implementation. Major challenges in implementation science are the lack of consensus regarding how to conceptualize context, how to examine context-specific effects on outcomes, and how to address context-related causal mechanisms that may affect implementation [26]. An inability to measure contextual factors limits generalizability of implementation strategies to different settings [27].

Within the Alliance, there are common implementation strategies such as practice facilitation and audit and feedback, which are utilized across all six research projects. Ongoing discussions and expressed needs highlight the importance of understanding how the context within each research setting, project, and country will impact the outcomes of implementation. To address this need, the RCC, the Data Harmonization Subcommittee, and the Dissemination and Implementation Science Subcommittee are working together to map mechanisms of action for the intervention and strategies being tested by the research teams. The implementation strategies being implemented by the research teams include information management, clinic workflow modification, audit and feedback, task shifting and strengthening, practice facilitation, development of educational materials, blood pressure control monitoring and medication use. Together, the subcommittees will work across the research projects to identify shared implementation, cost, and clinical outcomes measures that can be harmonized.

Our approach involves integrating emerging methodologies, such as Lewis’ causal pathway diagrams [28], with participatory group model building techniques to dissect the mechanisms underlying the implementation strategies [29]. The D&I core group collaborates with the study teams implementing interventions at various research sites across multiple sessions to achieve this objective. An initial phase specifies the implementation strategies using Proctor’s framework [18]. Subsequently, we engage in participatory mapping of these strategies using Lewis and colleagues’ Causal Pathway Diagrams [26]. Through this process, discussions are guided, facilitating the identification of moderating factors, preconditions for mechanism activation, and determinants at interpersonal, intrapersonal, and organizational levels. Continuing this mapping process for each research team, we anticipate gaining a comprehensive understanding of the relevant concepts and domains.

#### Effect of the COVID-19 pandemic on research activities

The initiation of the HLB-SIMPLe Alliance in 2020 coincided with the COVID-19 pandemic and presented challenges related to widespread restrictions of travel for in-person visits. The Alliance again had to postpone its inaugural in-person meeting scheduled for October 2022 to May 2023 due to an Ebola outbreak in host-country [30]. These events frustrated the Alliance’s desire for in-person interactions. The pandemic also introduced significant delays and disruptions for the six clinical trials. An illustration of this adaptation is evident in alterations to anti-retroviral therapy service delivery models from pre- to post-pandemic settings, thereby influencing the clinical trial landscape. Take, for instance, one of the research project sites where visits for anti-retroviral medication refills were typically scheduled monthly before the pandemic. However, in the post-COVID era, antiretroviral medication refills were often extended to every 3 to 6 months. This shift significantly curtailed the opportunities for collecting longitudinal clinical data, consequently impacting the duration of the intervention, and exacerbated stock-outs of antihypertensive medications. This health-systems change may ultimately facilitate higher medication adherence in PLHIV [31].

#### Site monitoring

One example of an adaptation implemented by the RCC was to perform remote regulatory site monitoring visits because of travel restrictions. The RCC collaborated with the Federal Team and research projects to ensure that regulatory visits were conducted within the necessary period to allow for timely UG3 (i.e., planning) to UH3 (i.e., active clinical trial) transition, and, to conduct annual regulatory site visits as needed throughout the active clinical trial. The purpose and agenda of the regulatory site monitoring visits include reviews of: (1) regulatory documents, (2) processes for safety reporting, (3) research protocol and manuals of operating procedures, (4) informed consent procedures, (5) data management and source data verification, and (6) implementation of appropriate quality assurance procedures. The site monitoring teams collaborated with research teams to address challenges uncovered in the regulatory monitoring visits.

The primary objectives of remote site monitoring is to ensure the rights and safety of human research participants are protected and the quality and integrity of clinical trial results. Furthermore, the monitoring procedures performed by the RCC aligns with federal policies and are based on the principle that the methods used in monitoring should be proportionate to the risks inherent in the trial. All HLB-SIMPLe Alliance awardee projects are conducting dissemination and implementation studies that include an effectiveness component and use evidence-based interventions associated with no more than minimal risks to research participants. Thus, future monitoring endeavors will continue to incorporate this flexible approach, reinforcing the safeguarding of participants’ rights and safety, while upholding the integrity and excellence of clinical trial outcomes.

#### Costing and cost analysis

Understanding the impacts and outcomes of costing on implementation strategy bundles is crucial. Given its significance to implementation fidelity, there was an expressed need within the alliance to develop a set of standardized instruments for collecting cost data in participating sites and to ensure that best practices regarding costing methods are shared across all sites. To address this need, the RCC identified an expert consultant from outside the organization who possesses in-depth supplementary expertise in health economics. The Steering Committee mandated the formation of a costing and health economics working group led by the expert consultant. This working group has served as a platform for discussion and shared learning on the health economics of intervention implementation.

Furthermore, the RCC, in collaboration with this working group, secured supplementary funding to collect cost data in the two HLB-SIMPLe research projects that initially did not include health economics in their research plan. This initiative aims to advance the science of costing the implementation of health interventions in LMICs and to bolster health economics capacity within the Alliance and beyond.

## Conclusions

The HLB-SIMPLe cooperative agreements bring together six implementation science research projects based in Botswana, Mozambique, Nigeria, South Africa, Uganda, and Zambia, a research coordinating center, and a United States Government Federal Team with the mission of providing a collaborative infrastructure that will facilitate the conduct of late-stage implementation research to optimally and sustainably deliver proven-effective prevention and treatment interventions for hypertension in PLHIV. To achieve its intended goals and performance objectives, the HLB-SIMPLe Alliance relies on a Steering Committee, subcommittees, and working groups, which are coordinated by the research coordinating center.

The RCC provides added shared learning opportunities and hence provides a valuable service to the group of six clinical trials and the Federal Team. Working together in the Alliance, the RCCs extends support and offers reliable and consistent venues for information sharing as sought by the research projects. The RCC offers an enhanced platform of meaningful internal and external communication activities, supporting dissemination and implementation science research activities, and offering a robust curricula of training and activities to build D&I research capacity in LMIC. Continuous and effective day-to-day collaboration between the RCC, research projects, Federal Team, and relevant collaborators has been critical in identifying opportunities for continuous improvement and effecting solutions to emerging challenges related to conducting research among large research teams in different countries.

## Data Availability

Not Applicable.

## Abbreviations

D&I: Dissemination and Implementation Research
DSMB: Data and Safety Monitoring Board
FIC: Fogarty International Center
HIV: Human Immunodeficiency Virus
HLB: SIMPLe–Heart, Lung, and Blood Co–morbiditieS Implementation Models in People Living with HIV
LMICs: Low–and Middle–Income Countries
MOOC: Massive Open Online Course
NCD: Non–communicable diseases
NIH: National Institutes of Health
NHLBI: National Heart, Lung, and Blood Institute
PLHIV: People Living with HIV
PROM: Project and Risks Operations Management Working Group
RCC: Research Coordinating Center
TSBM: Translational Science Benefits Model
